# Alcohol and Insanity

**Published:** 1892-05

**Authors:** 


### ALCOHOL AND INSANITY.

Some interesting facts have recently been developed in relation to the causation of insanity, through the efforts of a committee of physicians in San Francisco. The extent to which one cause, at least, acts, it would appear, has heretofore scarcely been duly appreciated. We have reference to alcohol, which may be held as directly or indirectly responsible for at least one-fourth of all the cases of insanity in this country.

Commenting on this subject the Journal of the American Medical Association says : There is no condition of the human mind that more strongly predisposes to insanity than protracted anxiety, which consists in a more or less intense desire for the accomplishment of a certain object, coupled with a constant fear of failure. The ranks of the great laboring classes, especially in this country, are full of individuals and families inspired with the anxious desire to improve their pecuniary, social, and educational condition, but whose baser passions and vicious indulgences either absorb so much of their earnings, or tempt them into such unlawful acts, as not only disappoint and disgrace the man, but send the arrows of long dreaded disappointment and despair deep into the minds of wife and children. One of the most prolific causes of intense and continued anxiety, ending often in both physical and mental ruin, is the use of alcoholic or fermented and distilled drinks, that takes directly from the earnings of labor in this country more than $500,000,000 annually, and brings the most intense and protracted anguish to the minds of many thousands of innocent parties.

